# Specific Age-Associated DNA Methylation Changes in Human Dermal Fibroblasts

**DOI:** 10.1371/journal.pone.0016679

**Published:** 2011-02-08

**Authors:** Carmen M. Koch, Christoph V. Suschek, Qiong Lin, Simone Bork, Maria Goergens, Sylvia Joussen, Norbert Pallua, Anthony D. Ho, Martin Zenke, Wolfgang Wagner

**Affiliations:** 1 Helmholtz-Institute for Biomedical Engineering, RWTH Aachen University Medical School, Aachen, Germany; 2 Department of Plastic and Reconstructive Surgery, Hand Surgery, Burn Center, RWTH Aachen University Medical School, Aachen, Germany; 3 Department of Medicine V, University of Heidelberg, Heidelberg, Germany; 4 Heidelberg Academy of Sciences and Humanities, Heidelberg, Germany; Deutsches Krebsforschungszentrum, Germany

## Abstract

Epigenetic modifications of cytosine residues in the DNA play a critical role for cellular differentiation and potentially also for aging. In mesenchymal stromal cells (MSC) from human bone marrow we have previously demonstrated age-associated methylation changes at specific CpG-sites of developmental genes. In continuation of this work, we have now isolated human dermal fibroblasts from young (<23 years) and elderly donors (>60 years) for comparison of their DNA methylation profiles using the Infinium HumanMethylation27 assay. In contrast to MSC, fibroblasts could not be induced towards adipogenic, osteogenic and chondrogenic lineage and this is reflected by highly significant differences between the two cell types: 766 CpG sites were hyper-methylated and 752 CpG sites were hypo-methylated in fibroblasts in comparison to MSC. Strikingly, global DNA methylation profiles of fibroblasts from the same dermal region clustered closely together indicating that fibroblasts maintain positional memory even after *in vitro* culture. 75 CpG sites were more than 15% differentially methylated in fibroblasts upon aging. Very high hyper-methylation was observed in the aged group within the *INK4A/ARF/INK4b* locus and this was validated by pyrosequencing. Age-associated DNA methylation changes were related in fibroblasts and MSC but they were often regulated in opposite directions between the two cell types. In contrast, long-term culture associated changes were very consistent in fibroblasts and MSC. Epigenetic modifications at specific CpG sites support the notion that aging represents a coordinated developmental mechanism that seems to be regulated in a cell type specific manner.

## Introduction

There is a growing perception that epigenetic modifications, such as DNA methylation and histone modification, play an important role for cellular senescence and aging of the organism [1]–[3]. CpG dinucleotides in the genomic DNA can be methylated at cytosine moieties. Upon replication the same methylation pattern is established on the newly synthesized DNA strand by DNA methyltransferase 1 (DNMT1) and thereby, the methylation pattern is inherited to both daughter cells. This inheritance of epigenetic modifications might provide an ideal mechanism for the regulation of progressive alterations in the course of aging [4].

Various studies have indicated, that the global DNA methylation level decreases upon aging in murine, rat and human tissues [5]–[7]. It was also shown that the 5-methylcytosine content decreased upon long-term culture of fibroblasts [8]. This led to the suggestion that the global loss of DNA methylation might be a result of passive demethylation as a consequence of a progressive loss of DNMT1a efficiency [9]. However, a number of specific loci become hyper-methylated during aging, such as the *INK4A/ARF/INK4b* locus, *the ribosomal gene cluster, estrogen receptor* (*ER*), *runt-related transcription factor 3* (*RUNX3*), *insulin growth factor II* (*IGF2*), *E-cadherin*, *c-fos* and others [1], [10]–[13]. These specific changes indicate that age-associated methylation changes are not simply based on random deterioration during ontogenic development although it is yet unknown how site-specific methylation changes are regulated.

We have recently analyzed age-associated DNA methylation changes in human mesenchymal stromal cells (MSC) [14]. These cells comprise multipotent precursors for mesodermal cell lineages such as osteocytes, chondrocytes and adipocytes and have therefore been coined as “mesenchymal stem cells”. Global methylation profiles were analyzed using the HumanMethylation27 BeadChip microarray allowing the determination of DNA methylation levels at 27,578 unique CpG sites within more than 14,000 promoter regions. Overall, methylation patterns of MSC were maintained throughout both, long-term culture and aging, whereas highly significant differences were observed at specific CpG sites. Notably, methylation changes as well as gene expression changes in MSC were overlapping in long-term culture *in vitro* and aging *in vivo* [14]–[17]. This supports the notion of replicative senescence and aging to represent related developmental processes, regulated by specific epigenetic modifications.

Distinct age-related phenotypes, such as wrinkle formation, hair graying and impaired wound healing, as well as the accessibility of samples from differently aged healthy donors make human skin an ideal model system for the analysis of age-related epigenetic changes [18], [19]. Dermal fibroblasts are important for skin architecture and extracellular matrix (ECM) synthesis and they are morphologically indistinguishable from MSC preparations. It is controversially discussed if immunomodulatory capacities and differentiation potential vary between the two cell types - some studies indicated that dermal fibroblasts display similar *in vitro* differentiation potential as MSC [20]–[22]. This might be explained by the finding that human dermal fibroblasts are composed of different subtypes with distinct gene expression profiles according to the anatomical site of origin [23]. With this in mind, we have isolated fibroblasts from different dermal regions to investigate age-associated changes in their DNA methylation profiles in comparison to MSC.

## Results

### Comparison of dermal fibroblasts with bone marrow MSC

**Figure 1:**
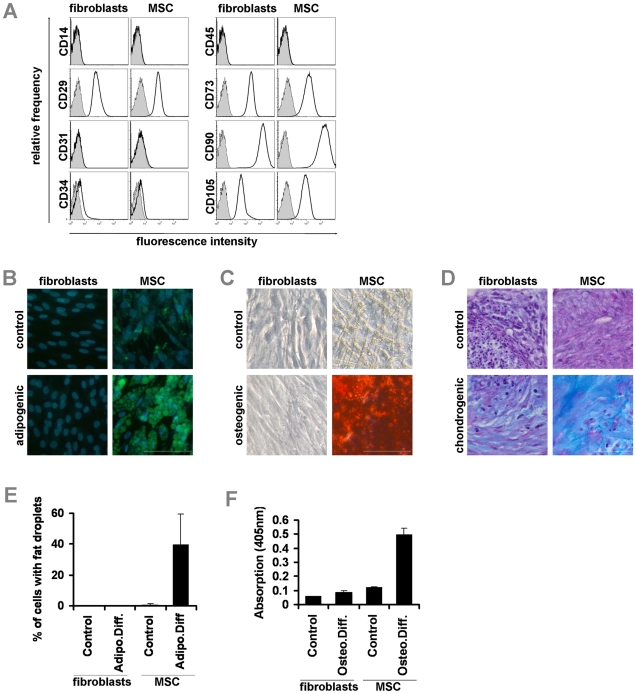
Molecular characterization of fibroblasts and MSC. Dermal fibroblast and mesenchymal stromal cells from bone marrow display a very similar immunophenotype (A). Adipogenic differentiation could not be induced in fibroblasts whereas fat droplet formation was observed in about 40% of the MSC upon differentiation (B,E; blue: DAPI; green: BODIPY; scale bar: 100 µm). Osteogenic differentiation was monitored by Alizarin Red staining of calcium phosphate precipitates and this could only be induced in MSC but not in fibroblasts (C,F; scale bar: 100 µm). Chondrogenic differentiation was analyzed by Alcian Blue staining of glucosaminoglycans in micromass culture and this was again only observed in MSC but not in fibroblasts (D; scale bar: 50 µm).

Human dermal fibroblasts were isolated from young (6–23 years old; 9 samples) and elderly donors (60–73 years old; 6 samples). Mesenchymal stromal cells were isolated from bone marrow of young (21–50 years old; 4 samples) and elderly donors (53–85 years old; 4 samples) as described in our previous work [14], [16]. Both cell types revealed a very similar spindle-shaped morphology and growth pattern. Furthermore, fibroblasts and MSC displayed the same and uniform immunophenotype for surface markers that are commonly used for the definition of MSC (CD14−, CD29+, CD31−, CD34−, CD45−, CD73+, CD90+, CD105+; Figure 1A). Simultaneous *in vitro* differentiation revealed that adipogenic differentiation could be induced in about 40% of the cells within MSC preparations, whereas hardly any fat droplet formation was observed in fibroblasts (n = 5, Figure 1B,E). Osteogenic differentiation induced calcific deposition only in MSC but not in fibroblasts (Figure 1C,F) and chondrogenic differentiation in micromass culture induced sulfated glycosaminoglycan rich matrix only in MSC but not in fibroblasts (Figure 1D). These results support the notion, that MSC but not fibroblasts comprise a multipotent subset of progenitor cells.

### DNA methylation changes between fibroblasts and MSC

**Figure 2:**
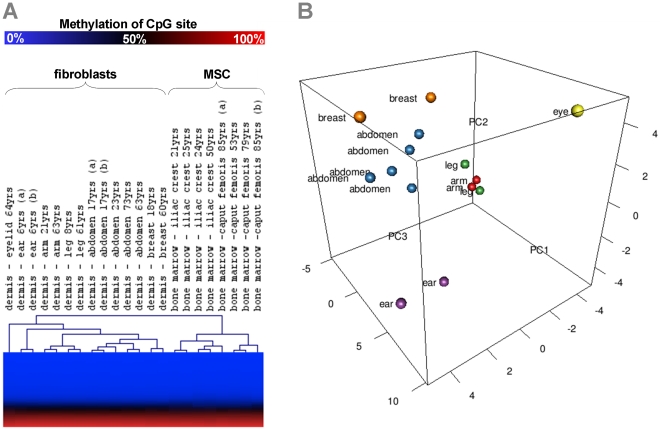
Relationship of DNA methylation profiles. DNA methylation profiles of fibroblasts and MSC were analyzed with the HumanMethylation27 BeadChip that facilitates simultaneous analysis of 27,578 unique CpG sites. Unsupervised hierarchical clustering (Euclidian distance) of all CpG sites separated fibroblasts and MSC in two different groups. Furthermore, cell preparations from the same anatomical site clustered together (A). Alternatively, we used principal components analysis (PCA) to visualize the relationship of fibroblasts from different dermal regions (B).

DNA methylation profiles of fibroblasts and MSC of early passage (passage 3 and passage 2 respectively) were compared using the HumanMethylation27 BeadChip microarray. Hierarchical cluster analysis clearly separated fibroblasts and MSC into two different groups (Figure 2A) and histograms of P-values demonstrate a skew towards 0 which supports the notion of a high number of significant differences (Figure S1A). Various CpG sites revealed differences in average beta greater than 0.15 (referred to as more than 15% difference in methylation level) between fibroblasts and MSC and additionally passed a second filter criterion of highly significant changes (*P*<0.001 with limma moderated T-test): 766 CpG sites were hyper-methylated in fibroblasts whereas 752 CpG sites were hypo-methylated. These CpG sites corresponded to 521 and 499 non-redundant genes that were categorized by Gene Onthology classification. Hyper-methylated CpG sites in fibroblasts were significantly over-represented in genes of categories that mediate development, immune response and hormone secretion. Hypo-methylated genes were rather involved in immune response, defense response and wound healing (Figure S2). Taken together, fibroblasts and MSC differ in their epigenetic profiles and this may result in their dissimilar differentiation potential and immunomodulatory function.

### Epigenetic positional memory of fibroblasts

It was striking that DNA methylation profiles of fibroblasts from the same dermal region such as ear, arm, breast, abdomen or leg clustered always closely together in hierarchical cluster analysis (Figure 2A). To depict differences that lead to the clustering, we selected 3182 CpG sites that might be differentially methylated among anatomical sites (*P*<0.001; limma moderated F-statistic). These CpG sites were then subjected to a heatmap (Figure S3). Differences in fibroblasts from different anatomical sites are further supported by principal components analysis (PCA; Figure 2B). Such positional memory might be governed by epigenetic modification of homeobox containing or *HOX-genes* that have been shown to be important for patterning the primary and secondary axes [24], [25]. Hierarchical clustering of 85 CpG sites represented on the microarray that are associated with the four *HOX* clusters revealed methylation changes that correlate with different anatomical sites (Figure S4). Thus, fibroblasts and MSC maintain positional information in their DNA methylation profile even after culture expansion for three passages.

### Age-associated DNA methylation changes in fibroblasts

**Figure 3:**
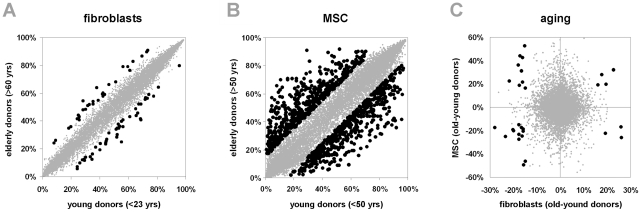
Age-associated methylation changes in fibroblasts and MSC. Mean DNA methylation of 27,578 CpG sites was compared between fibroblasts from young donors (<23 years, eight samples) and from elderly donors (>60 years; six samples) by scatter plot analysis. 75 CpG sites revealed more than 15% age-associated differential methylation (indicated as black spots; A). Scatterplot analysis of MSC from young (<50 years, four samples) and elderly donors (>50 years, four samples) demonstrated that 1060 CpG have more than 15% age-associated DNA methylation changes (B). Comparison of fibroblasts revealed that 30 CpG sites were overlapping differentially methylated upon aging in MSC and fibroblasts (black spots; C). This is highly significantly more than expected by chance alone but differential methylation often occurred in opposite directions.

Comparison of DNA methylation profiles in fibroblasts derived from young (<23 years old) or elderly donors (>60 years old) revealed that 75 CpG sites change their methylation level more than 15% upon aging (Figure 3A). However, none of these were highly significant using the stringent criteria as mentioned above (*P*<0.001; limma moderated T-test). This might be due to the relatively small sample number or other parameters which may impact differential methylation. Alternatively, we performed pair wise comparisons with fibroblasts from the same dermal location to exclude bias by sampling from different anatomical sites, and these results were very similar (data not shown). Only skin samples from female donors were utilized to avoid DNA methylation differences due to dose compensation of sex chromosomes [19]. On the other hand, there is a tendency towards zero in the histogram of P-values indicating that there might be specific age-associated changes (Figure S1B,C). We used rank product (RankProd) test as alternative non-parametric method to generate adjusted p-values and 257 CpG sites reached a significance level below 0.05. These included 31 CpG sites of the 75 CpG sites with methylation changes of more than 15% (Table S1).

Genes with CpG sites that revealed the highest age-associated hyper-methylation included *sperm associated antigen 7* (*SPAG7*; 26%), *cyclin-dependent kinase inhibitor 2B* (*CDKN2B*; 26%) and *Stannin* (*SNN*; 20%). In contrast, genes with hypo-methylated CpG sites included *aldehyde dehydrogenase 1A1* (*ALDH1A1*; −30%); *complement factor D* (*CFD*; −22%); *WNT1 inducible signaling pathway protein 2* (*WISP2*; −18%) and *cyclin-dependent kinase 5, regulatory subunit 1* (*CDK5R1*; −17%). Subsequently, we have tested how differential DNA methylation is reflected on gene expression level: quantitative real time PCR of 7 genes demonstrated that in tendency, hypomethylation upon aging is associated with higher gene expression and this was significant for *WISP2*, *ALDH1A1* and *CFD* (Figure S5).

Other authors indicated that the global methylation level decays upon aging. Therefore, we reanalyzed non-quantil normalized raw-data and there was a slight decline of the methylation level upon aging in fibroblasts (Figure S6). The 90-quantil of methylation level in this boxplot was 80.5% in fibroblasts from younger donors whereas it was 78.5% in fibroblasts from elderly donors (*P* = 0.02). In contrast, global age-associated hypomethylation was not significant in MSC (*P* = 0.93). 1060 CpG sites were more than 15% differentially methylated in MSC of young and elderly donors [14] (Figure 3B). There was no linear correlation in age-associated changes in fibroblasts and MSC. Strikingly, 30 CpG sites were in the overlap of CpG sites with age-associated changes in fibroblasts and MSC (Figure 3C, Table S2). Thus, 40% of age-associated changes in fibroblast CpG sites were also differentially methylated in MSC and hypergeometric distribution revealed that this association is highly significant (*P*<10^−25^). Several CpG sites that are hyper-methylated upon aging of fibroblasts were hypo-methylated upon aging of MSC and *vice versa*.

**Figure 4:**
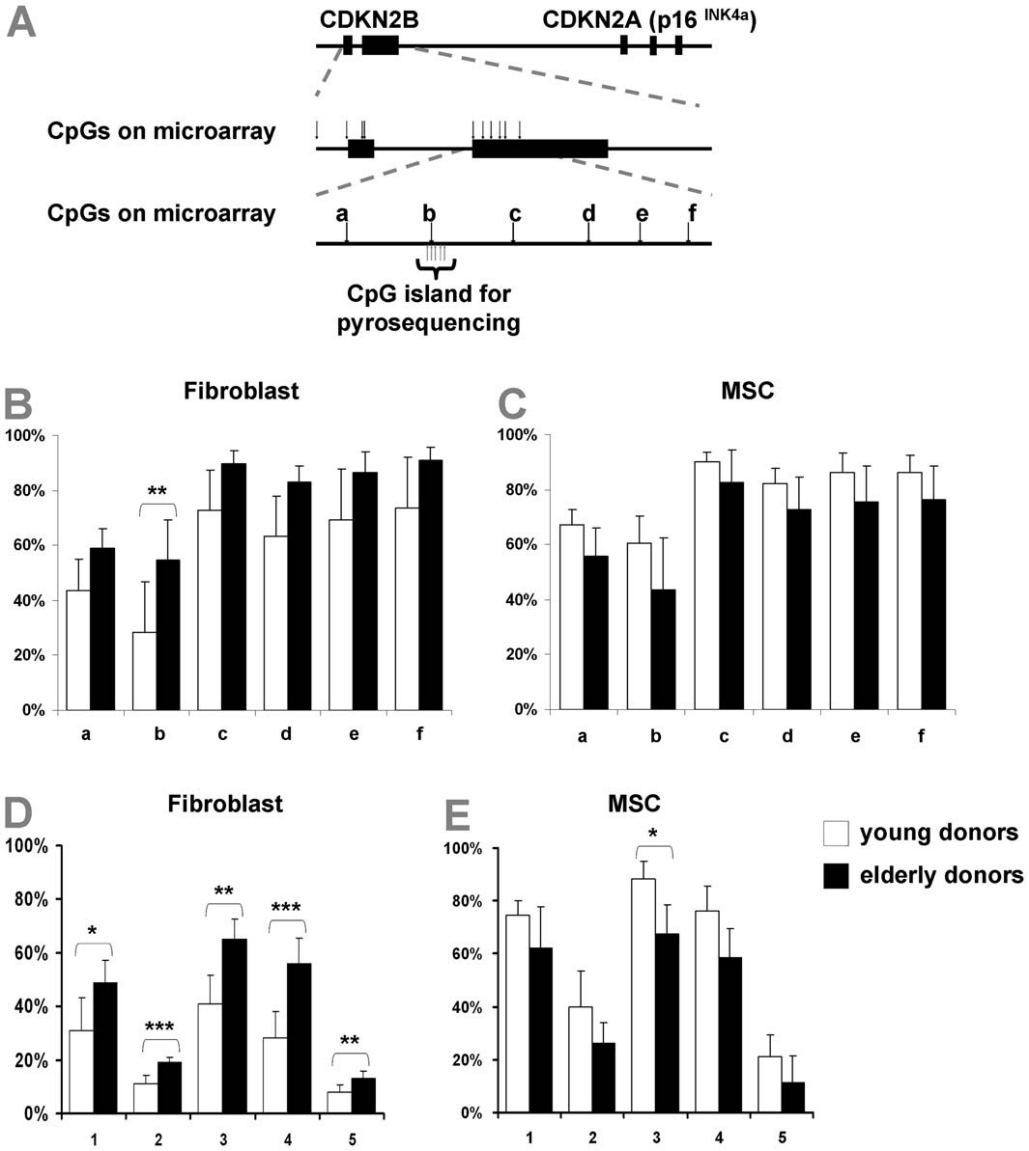
Age-associated DNA methylation changes in *CDKN2B*. *Cyclin-dependent kinase inhibitor 2B* (*CDKN2B*) belongs to the *INK4A/ARF/INK4B* locus and six CpG sites within the second exon are represented by the microarray (A). All of these were hyper-methylated upon aging in fibroblasts (B) and they were all hypo-methylated in MSC (C). Pyrosequencing of five neighbouring CpG sites in this region verified significant hyper-methylation upon aging in fibroblasts (D) and hypo-methylation in MSC (E). * P<0.05; ** P<0.01; *** P<0.001).

Age-associated methylation changes were analyzed in more detail for *CDKN2B* that is part of the *INK4A/ARF/INK4b* locus. Notably, all 6 CpG sites within the second exon of *CDKN2B* represented on the HumanMethylation27 BeadChip microarray were hyper-methylated upon aging in fibroblasts, whereas they were hypo-methylated in MSC. These age-associated DNA methylation changes were exemplarily validated by pyrosequencing and this method revealed very similar age-associated methylation changes in five neighboring CpG sites (Figure 4). Taken together, there are some age-associated DNA methylation changes at specific CpG sites in fibroblasts and MSC but they are often regulated in opposite directions in a cell type dependent manner.

### Replicative senescence affects DNA methylation patterns

**Figure 5:**
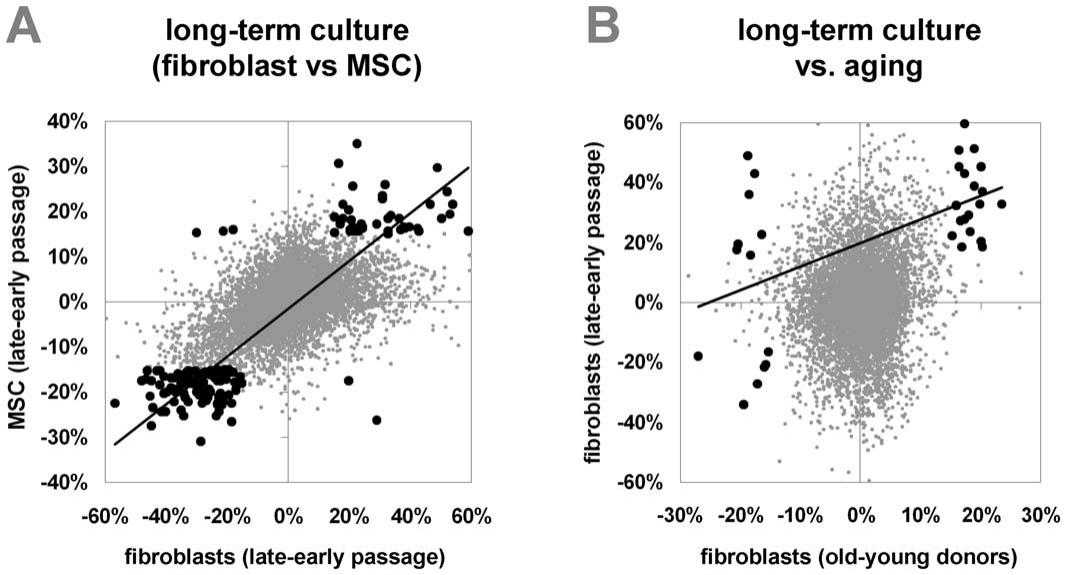
Long-term culture-associated methylation changes in fibroblasts and MSC. Differential methylation between passage 3 and passage 21 of fibroblasts (6 year old donor) was plotted against differential DNA methylation data upon long-term culture of MSC that has been described before [14] (A). Black spots demonstrate CpG sites with differential methylation of more than 15% upon long-term culture of both cell types and these showed a very good correlation in long-term culture associated changes in fibroblasts and MSC (R = 0.764). We have also compared long-term culture-associated DNA methylation changes and age-associated changes in fibroblasts and they display only moderate correlation (R = 0.337; B).

Long-term culture of MSC has impact on proliferation, cell size and differentiation potential [26] and we have recently demonstrated that this is associated with reproducible and highly significant DNA methylation changes at specific CpG sites [14]. To determine if similar epigenetic modifications are induced by long-term culture of fibroblasts, we have compared DNA methylation profiles of passage 3 and passage 21 of fibroblasts (6 year old donor, othoplastic). Comparison of long-term culture associated DNA methylation changes in fibroblasts and MSC revealed a striking correlation in CpG sites that were more than 15% differentially methylated in both cell types (P<10^−31^, chi-square analysis of 161 CpG sites; Figure 5A). Thus, replicative senescence induces similar hyper-methylation and hypo-methylation at specific CpG sites in fibroblasts and MSC. Furthermore, there was a significant correlation between DNA methylation changes in fibroblasts upon long-term culture *in vitro* and aging *in vivo* (P = 0.001, chi-square analysis of 32 CpG sites with more than 15% methylation changes in both comparisons; Figure 5B).

## Discussion

Fibroblasts are diverse with various functions in different dermal regions [23]. This diversity is also reflected in global DNA methylation patterns: epigenetic modifications at specific CpG sites provide a positional memory of the sampling location and there are age-associated DNA methylation changes that may contribute to aging of the skin.

The question, how to distinguish between fibroblasts and MSC, is not trivial. MSC raise high hopes for cellular therapy and regenerative medicine and they are concurrently tested in various clinical trials [27]. Despite intensive research over the last decade, there is still no molecular definition of multipotent subsets. MSC and fibroblasts are very similar in cell size, morphology, growth pattern and immunophenotype [28]–[30]. Traditionally, MSC have been defined by their multipotent differentiation potential [31], [32] but this demarcation has lost its clarity since it has been shown, that human dermal fibroblasts may also comprise multipotent cells with adipogenic, osteogenic and chondrogenic differentiation potential [33], [34]. However, for the samples used in this study, adiopogenic and osteogenic differentiation could only be induced in MSC but not fibroblasts. Hence, the difference in DNA methylation profiles between fibroblasts and MSC may also arise from a specific epigenetic make up of the multipotent subset within MSC. Further MSC preparations isolated under different culture conditions and other cell types need to be considered to determine epigenetic modifications that are characteristic for adult stem cells [32].

Reciprocal epithelial-mesenchymal interactions shape site-specific development of the skin. Recombination experiments in chick suggested that dermal signals dictate positional identity of epidermal differentiation: for example transplantation of wing epithelium to leg mesenchyme transforms the development of feathers to the development of scales [35], [36]. Various studies have addressed the anatomic diversity of human fibroblasts on the gene expression level [23], [36], [37]. These datasets demonstrated the association of global gene expression profiles with the site of fibroblast origin and they were related to three anatomic divisions: anterior - posterior, proximal - distal and dermal *versus* non-dermal. It has also been shown, that site-specific HOX expression in fibroblasts maintains features of the embryonic HOX pattern over decades *in vivo* and over numerous cell divisions *in vitro* [36]. In this study, we demonstrate that fibroblasts also maintain an epigenetic positional memory even after culture expansion for three passages. Hierarchical clustering of CpG sites related to HOX genes revealed a separation of fibroblasts from the top half (anterior) *versus* the bottom half (posterior) of the human body. Recently it has been shown, that variably methylated regions (VMRs) exist in the genome which are highly variable in DNA-methylation among individuals [38]. Several of these were in developmental genes including HOX genes and therefore inter-individual differences in fibroblast CpG methylation may act as a potential contributor to the site-dependent structure. Thus, a larger number of samples from various regions will be necessary to gain further insight into the systematics of positional determination by epigenetic modifications. This might also be useful for forensic analysis of tissue samples of unknown origin.

Various molecular mechanisms have been implicated in aging including telomere-shortening, accumulation of mutations, oxidative stress and alteration of molecular pathways [39]. Decreases in the global level of methylation along with a concomitant increase in promoter methylation has become a hallmark of age-associated epigenetic changes [5]–[7], [40]. Decrease of the 5-methylcytosine content upon aging has been demonstrated almost 40 years ago in the brain, heart and spleen of rats [6] and there is even evidence, that the global methylation level changes upon aging in plants [41]. In this study, we observed only a moderate age-associated hypo-methylation in fibroblasts. This might be due to the bias for promoter regions within the 27,578 CpG sites represented on the microarray whereas it has been shown, that hypo-methylation upon aging occurs predominantly in specific interspersed repetitive sequences (IRSs) [42]. Age-associated DNA methylation changes appear to be more complex as methylation of specific CpG sites reveal directional perturbations [12], [43], [44]. For example, Christensen et al. provided clear evidence of tissue–specific methylation changes related to aging [43].

The *INK4a/ARF/IN*K4b locus (also known as *CDKN2A* and *CDKN2B*) plays a pivotal role in aging and cancer [45]. Gene expression of *p16^INK4A^* (*CDKN2A*) increases with age and it has even been proposed as a biomarker of aging [46]. Furthermore, functional studies in hematopoietic stem cells, neuronal stem cells and pancreatic islets have suggested, that p16^INK4A^ is one cause of aging by down-regulation of self-renewal potential of adult stem cells [46]–[48] and reprogramming towards induced pluripotent stem cells is critically dependent on the repression of *p19^ARF^* in mice and *p16^INK4A^* in humans [49]. In this study, we observed the second-highest hyper-methylation upon aging of fibroblasts in *CDKN2B*. However, it is intriguing that the same CpG sites are hypo-methylated upon aging in MSC.

Overall, age-associated DNA methylation changes occurred at similar CpG sites upon aging in fibroblasts and MSC, but they were often regulated in different directions. This may underline the functional difference between the two cell types and the involvement of different mechanisms for cellular aging. It is also conceivable, that epigenetic modifications are differently regulated by the diverse cellular microenvironments in dermis and bone marrow. In our analysis, ages in fibroblasts and MSC were not matched and aging in MSC might be biased by the different available sources of bone marrow in young and elderly donors as described before [14]. All the more, the significant interrelation of age-associated methylation changes in fibroblasts and MSC is noteworthy. Recently, polycomb group protein targets have been shown to become far more likely methylated with age [13]. Furthermore, hyper-methylation occurs predominantly at bivalent chromatin domain promoters in CD4+ T-cells and CD14+ monocytes [12]. These are developmentally regulated genes harboring both, activating (H3K4me3) and inactivating (H3K27me3) histone marks in embryonic stem cells [50]–[52]. The bivalent epigenetic modifications raise the possibility of age-associated genes being poised in a bipotential state, which may be resolved differently in various cell lineages. This might explain the opposing age-associated DNA methylation changes in different tissues [43] and between fibroblasts and MSC.

Replicative senescence upon long-term culture is not an inevitable fate of all cells. Embryonic stem cells as well as induced pluripotent stem cells do not show signs of replicative senescence in long-term culture [53]. Loss of pluripotency seems to be accompanied with limitation of replicative live span and this might be governed by specific epigenetic modifications. We have previously demonstrated continuous changes in gene expression profiles of MSC upon long-term culture [15], [54] and observed a moderate but significant concordance in the expression profiles upon long-term culture *in vitro* and aging *in vivo* [16]. Furthermore, we have shown that long-term culture of MSC induces hyper-methylation and hypo-methylation at specific CpG sites and that these epigenetic modifications are also related to those between MSC from young and elderly donors [14]. Here, we have exemplarily analyzed DNA methylation changes upon long-term culture of fibroblasts and there was a striking concordance of senescence-associated methylation changes in fibroblasts and MSC. Additional fibroblast samples are currently culture-expanded to further discern long-term culture associated DNA methylation changes. This might facilitate identification of specific CpG sites for monitoring the replicative lifespan in cellular products for therapeutic application [17]. Notably, there was also a significant association of methylation changes upon long-term culture and aging in fibroblasts. This indicates that both mechanisms might be regulated by similar epigenetic modifications.

Two fundamental hypothesis are currently discussed to account for aging: it might be evoked by stochastic or random, accidental events, or it is a result of a purposeful program driven as developmental process [55]. Most likely, interplay of both mechanisms promotes aging at various levels. However, the highly reproducible epigenetic modification at specific CpG sites in developmental genes argues for a coordinated developmental mechanism. This study demonstrates, that fibroblasts and MSC maintain their positional memory as well as age-associated epigenetic modifications even after *in vitro* culture. Age-associated modifications seem to be regulated in a cell type specific manner. Further research on other characterized cell preparations and larger numbers of samples are important to gain insight into the mechanisms that control DNA methylation at specific sites in the genome.

## Materials and Methods

### Isolation of primary cells

Human dermal fibroblasts were isolated from skin samples donated by young (6–23 years) and elderly (60–73 years) female patients undergoing surgical interventions. All samples were taken after written consent, using guidelines approved by the Ethic Committee on the Use of Human Subjects at the University of Aachen. Skin samples were treated with dispase II (Roche Diagnostics, Mannheim, Germany) for 12 hours at 4°C to separate the dermis from the epidermis. The dermis was digested with 0,2% collagenase and 1,5% BSA in collagenase buffer (100 mM HEPES, 120 mM NaCl, 50 mM KCl, 1 mM CaCl_2_, 5 mM Glucose) for 45 minutes. Dermal remnants were removed by filtering the digest through a 100 µm nylon strainer (Falcon, Becton Dickinson [BD], San Jose, USA). The cells were subsequently washed and expanded in standard medium consisting of DMEM (PAA; 1 g/L glucose) supplemented with glutamine (PAA), penicillin/streptomycin (PAA) and 10% fetal calf serum (Biochrom, Berlin, Germany) in a humidified atmosphere at 5% CO_2_.

Mesenchymal stromal cells were isolated from human bone marrow after written consent using guidelines approved by the Ethic Committee on the Use of Human Subjects at the University of Heidelberg as described before [16]. In brief, MSC were isolated from bone marrow aspirates from the iliac crest of young healthy donors for allogeneic transplantation or from the caput femoris after hip fracture of elderly donors. MSC were culture-expanded in culture medium M1 as previously described [16], [30].

### Immunophenotypic analysis

Surface marker expression was analyzed on a FACS canto II (BD) upon staining with the following antibodies as described before [16]: CD14-allophycocyanin (APC, clone M5E2, BD), CD29-phycoerythrin (PE, clone MAR4, BD), CD31-PE (clone WM59, BD), CD34-APC (clone 8G12, BD), CD45-APC (clone HI30, BD), CD73-PE (clone AD2, BD), CD90-APC (clone 5E10, BD), CD105-fluorescein isothiocyanate (FITC, clone MEM-226, ImmunoTools, Friesoythe, Germany).

### In vitro differentiation

Osteogenic, adipogenic and chondrogenic differentiation of fibroblasts and MSC was simultaneously performed under the same differentiation conditions as described before [15], [56]. After three weeks, osteogenic differentiation was analyzed by Alizarin Red staining and quantified with a method based on acetic acid extraction and neutralization with ammonium hydroxide [57]. A Tecan infinite M200 plate reader was employed to measure the absorbance at 405 nm.

Adipogenic differentiation was stained with the green fluorescent dye BODIPY (4,4-difluoro-1,2,5,7,8-pentamethyl-4-bora-3a,4a-diaza-s-indacene) and counter-stained with DAPI (4′,6-Diamidin-2-phenylindol; both Molecular Probes, Eugene, Oregon, USA). Fluorescence microscopic pictures were always taken from four randomly chosen areas with a Leica DM IL HC microscope (Leica, Wetzlar, Germany). Cell nuclei and BODIPY positive cells were counted with ImageJ software (rsbweb.nih.gov/ij/) to determine the percentage of cells that differentiated towards adipogenic lineage as described before [58].

Chondrogenic differentiation was induced in micromass culture for three weeks. Subsequently the pellets were fixed with 10% formalin and paraffin embedded. 1 µm sections were stained with Alcian blue in combination with PAS (Periodic acid-Schiff) in an automated slide stainer and photo-documented.

### DNA isolation

Genomic DNA was isolated from 10^6^ cells using the QIAGEN DNA Blood Midi-Kit. DNA quality was assessed with a NanoDrop ND-1000 spectrometer (NanoDrop Technologies, Wilmigton, USA) and gel electrophoresis. 600 ng DNA were subsequently bisulfite converted using the EpiTect Bisulfite Kit (Qiagen, Hilden, Germany).

### DNA methylation profiling

DNA methylation profiles were analyzed using the HumanMethylation27 BeadChip (Illumina, San Diego, USA) as described before [14]. About 200 ng of bisulfite converted DNA were applied per BeadChip according to the manufacturer's instructions. During hybridization, the DNA molecules anneal to two different bead types with locus-specific DNA oligomers - one corresponds to the methylated (C) and the other to the unmethylated (T) state. Allele-specific primer annealing is followed by single-base extension using DNP- and Biotin-labeled ddNTPs. After extension, the array is fluorescently stained, scanned, and the intensities of the unmethylated and methylated bead types measured. Hybridization and initial data analysis with the BeadStudio Methylation Module was performed at the DKFZ Gene Core Facility in Heidelberg. The precise distribution of samples across the beadchips is presented in Table S3. The complete CpG methylation values have been deposited in NCBIs Gene Expression Omnibus (GEO, http://www.ncbi.nlm.nih.gov/geo/) and are accessible through GEO Series accession number GSE22595. *(Hyperlink for review:* http://www.ncbi.nlm.nih.gov/geo/query/acc.cgi?token=rbsjfkqoqoukstc&acc=GSE22595 *)*.

DNA methylation values, described as beta values, are recorded for each locus in each sample. Background normalized raw data were only used to compare the overall methylation level in samples from young and elderly donors. For all other analysis, quantile normalization of the raw data was performed to minimize the chip effect [59]. Hierarchical clustering by Euclidian distance was performed using the MultiExperiment Viewer (MeV, TM4) [60]. Principal components analysis (PCA) was calculated with prcomp in R package stats. For analysis of age-associated DNA methylation changes we have calculated mean methylation of samples from young and elderly donors. To focus on biologically relevant CpG sites we have selected those with differences in average beta value greater than 0.15 (more than 15% difference in methylation level). Standard deviation in DNA-methylation measurements are not uniformly distributed across the range of beta values – it has a parabolic shape with the maximum peak around 0.5 [61]. Smaller changes at the two extremes of the beta-scale might also be important but they are less likely to be biologically relevant, more difficult to validate and using the 15% methylation threshold reduces beadchip effects which might exist despite quantile normalization.

To further select for significantly differentially methylated CpG sites we used a false discovery (FDR) approach using limma moderated T-test, which is based on an empirical Bayes moderation approach [62]. For comparison of fibroblasts and MSC we used very stringent parameters with 15% methylation changes and adjusted p-value (q-value) <0.001 [61]. However, these criteria were too stringent for analysis of aging in fibroblast because of the relatively small number of samples and therefore, we used an additional non-parametric method named RankProd with q-value <0.05. This method shared many CpG sites with the above mentioned cut-off of 15% methylation change. Genes associated with the differentially methylated CpG sites were classified by GeneOntology analysis using GoMiner software (http://discover.nci.nih.gov/gominer/).

### Pyrosequencing

Methylation changes in *CDKN2B* were validated by pyrosequencing as described before [14]. Pyrosequencing was performed at Varionostic GmbH (Ulm, Germany).

### Quantitative real-time PCR analysis

Expression of 7 differentially methylated genes was analyzed by real-time quantitative PCR (qRT-PCR) using the StepOne™ Instrument (Applied Biosystems, Applera Deutschland GmbH, Darmstadt, Germany). Total RNA was isolated and reversely transcribed as described in our previous work [16]. Primers were obtained from Metabion (Martinsried, Germany) (Table S4). QRT-PCR reactions were performed with the Fast SYBR® green PCR master mix in a MicroAmp optical 96-well reaction plate according to the manufacturer's instructions (Applied Biosystems). Gene expression levels were normalized to GAPDH expression.

### Statistics

All results are expressed as mean ± standard deviation. To estimate the probability of differences we have adopted the two-sided Student's T-test. Probability value of *P*<0.05 denoted statistical significance. Probability that age-associated methylation changes are related in different datasets was estimated by hypergeometric distribution, and linear correlation was determined by Pearson correlation coefficient. Representation of differentially methylated genes in functional categories was analyzed by Fischer's Exact p-value.

## Supporting Information

Figure S1 **Histograms of limma moderated T-test raw p-values.** For comparison of fibroblasts of young *versus* elderly donors, we performed nonspecific filtering to exclude the CpG sites with low variation by applying nsFilter function in genefilter package in R/Bioconductor. We then compared the histograms of raw p-values before and after filtering. This shows that the non-specific step can improve our analysis.
(JPG)

Figure S2 **Gene Onthology analysis of genes with differentially methylated CpG sites between fibroblasts and MSC.**
(TIF)

Figure S3 **Heat map of CpG sites which lead to clustering according to sampling location.** Samples were classified according to their sampling location (iliac crest, caput femoris, eyelid, ear, arm, leg, breast and abdomen). 3182 CpG sites were selected that might be differentially methylated (*P*<0,001; limma moderated F-statistic). These CpG sites were then subjected to a heatmap. Each CpG was normalized to zero and unit standard deviation.
(JPG)

Figure S4 **Hierarchical clustering of 85 CpG sites associated with the four** ***HOX*** **clusters.**
(TIF)

Figure S5 **qRT-PCR of differentially methylated genes in fibroblasts of young and elderly donors.**
(TIF)

Figure S6 **Boxplot of methylation level in raw data of beadchips.**
(TIF)

Table S1 **Age-associated methylation changes in fibroblasts.**
(DOC)

Table S2 **Age-associated changes in fibroblasts and MSC.**
(DOC)

Table S3 **Distribution of all the samples across the beadchips.**
(DOC)

Table S4 **Primer sequences for qRT-PCR.**
(DOC)
