# Supplementary figures and images for: Specific Age-Associated DNA Methylation Changes in Human Dermal Fibroblasts
Source: PLoS One. 2011 Feb 8;6(2):e16679. doi: 10.1371/journal.pone.0016679 (PMC3035656; doi:10.1371/journal.pone.0016679)

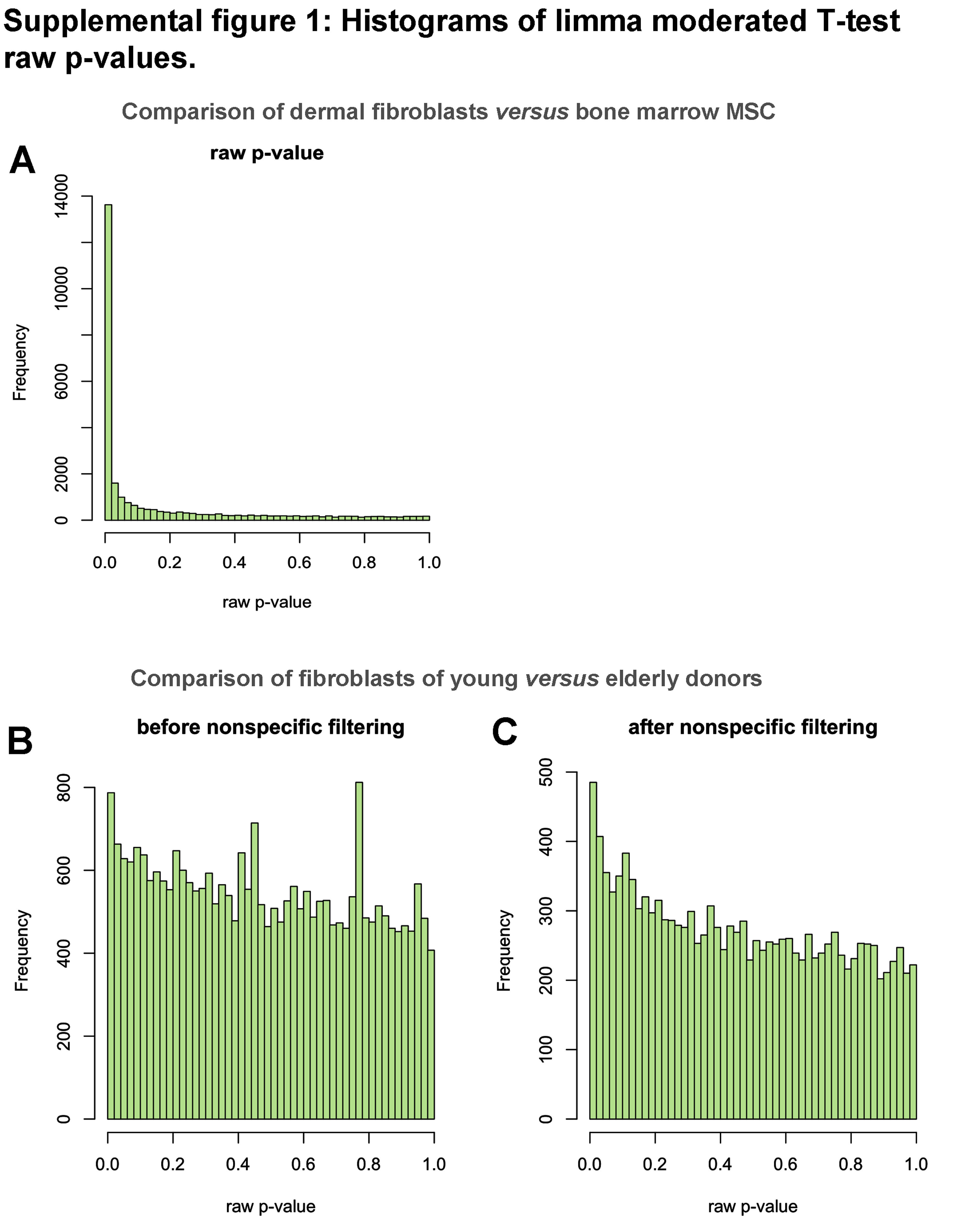

Supplement: Figure S1 — Histograms of limma moderated T-test raw p-values. For comparison of fibroblasts of young versus elderly donors, we performed nonspecific filtering to exclude the CpG sites with low variation by applying nsFilter function in genefilter package in R/Bioconductor. We then compared the histograms of raw p-values before and after filtering. This shows that the non-specific step can improve our analysis. (JPG) [file pone.0016679.s001.jpg]

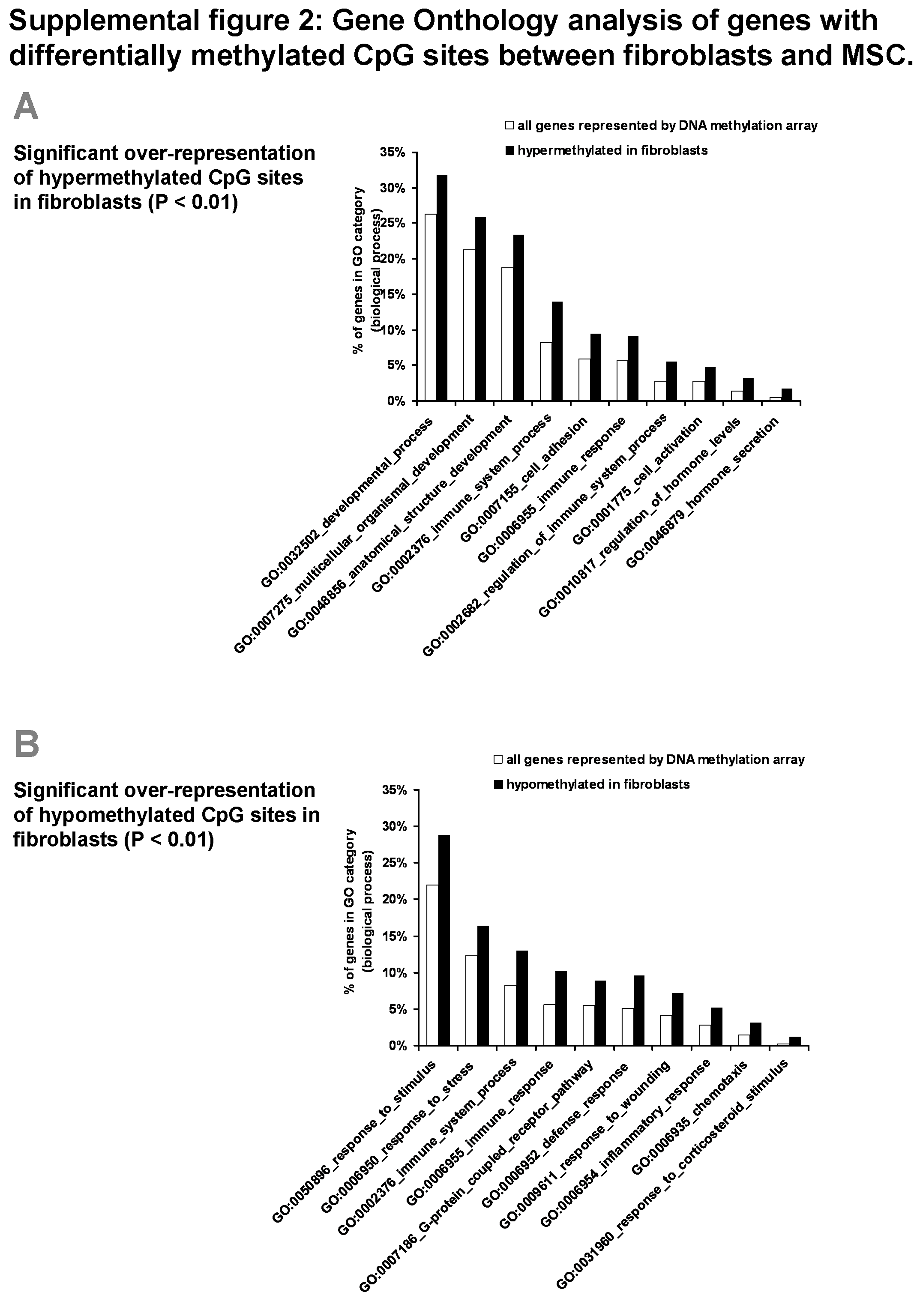

Supplement: Figure S2 — Gene Onthology analysis of genes with differentially methylated CpG sites between fibroblasts and MSC. (TIF) [file pone.0016679.s002.tif]

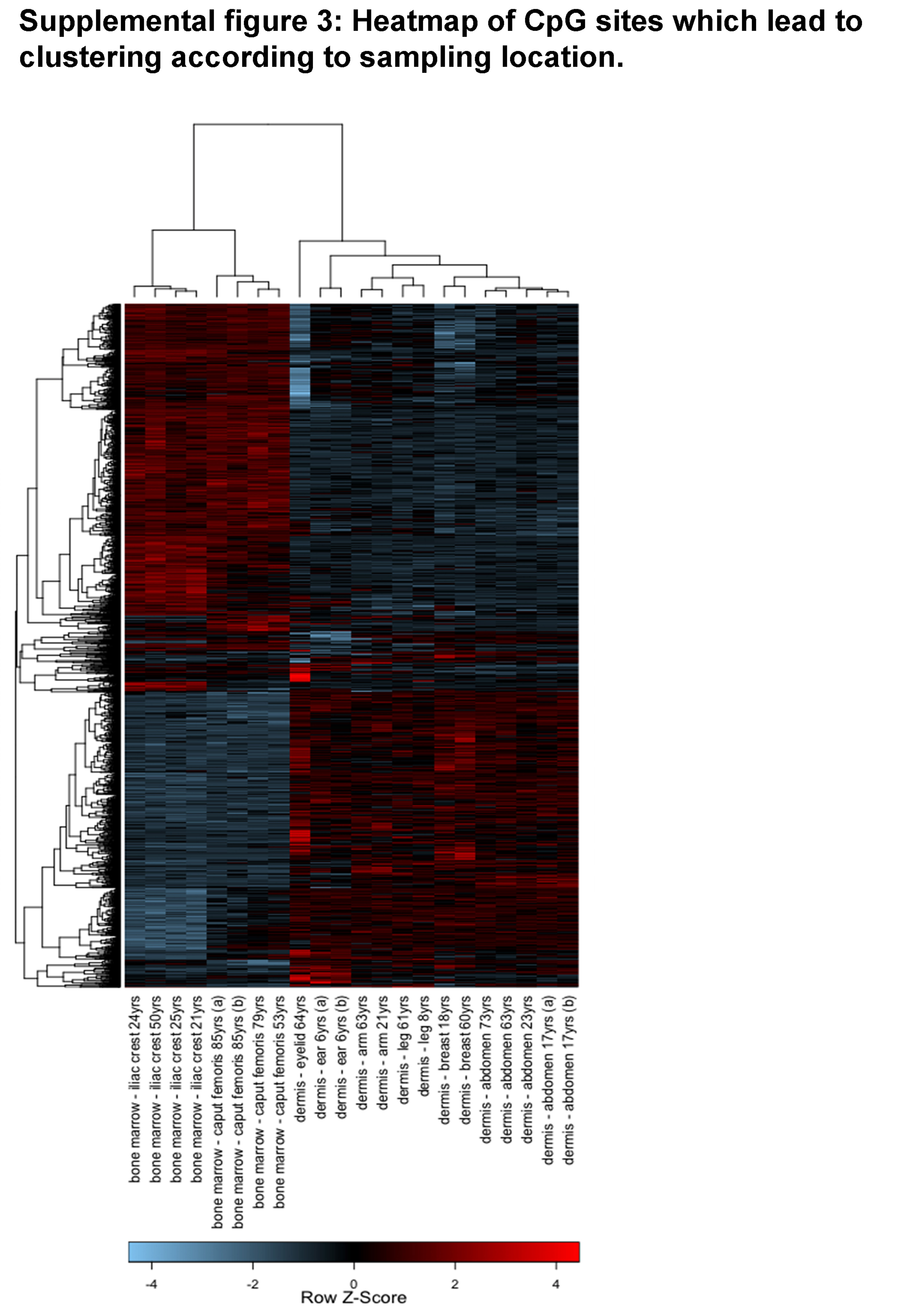

Supplement: Figure S3 — Heat map of CpG sites which lead to clustering according to sampling location. Samples were classified according to their sampling location (iliac crest, caput femoris, eyelid, ear, arm, leg, breast and abdomen). 3182 CpG sites were selected that might be differentially methylated (P<0,001; limma moderated F-statistic). These CpG sites were then subjected to a heatmap. Each CpG was normalized to zero and unit standard deviation. (JPG) [file pone.0016679.s003.jpg]

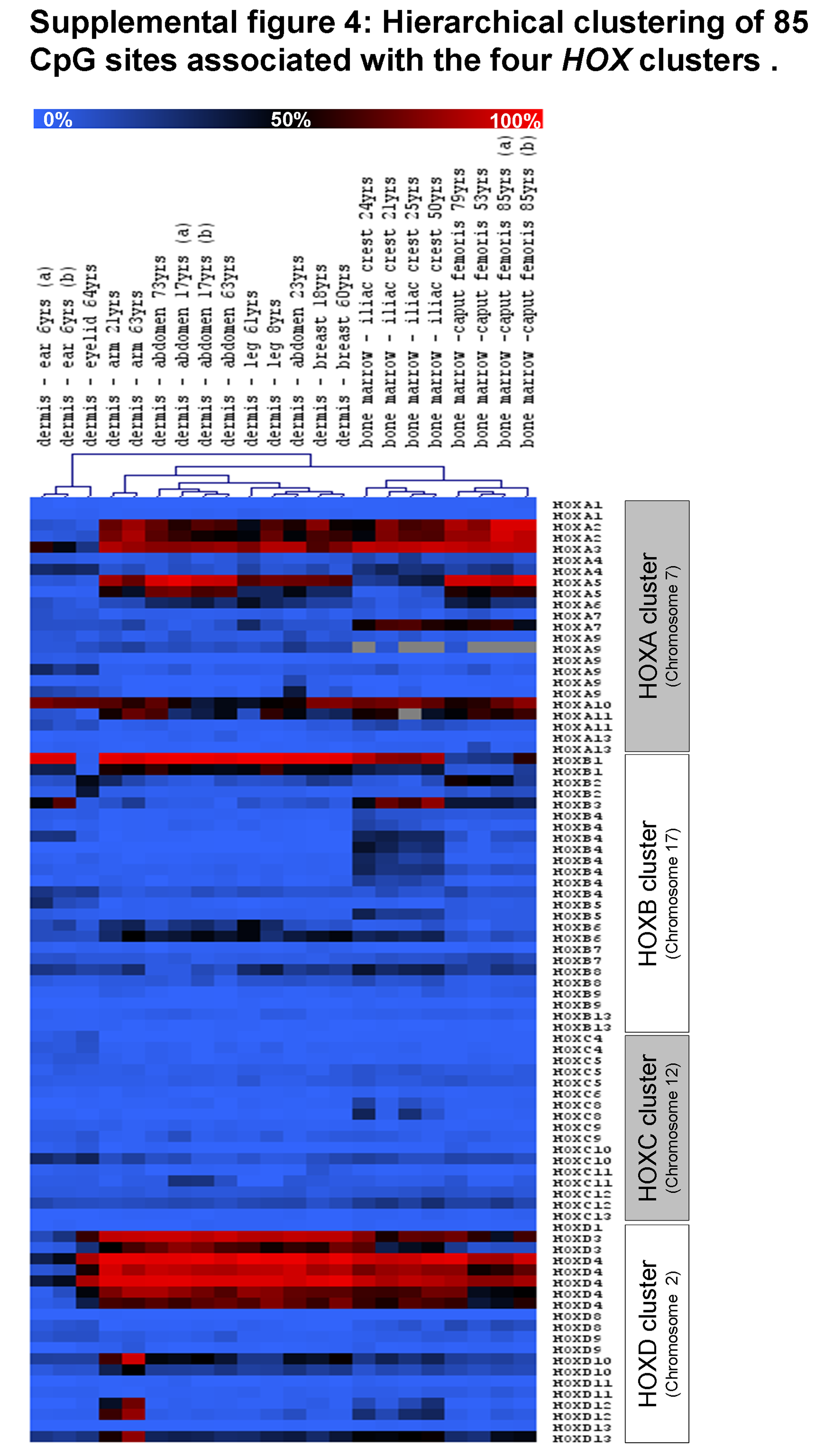

Supplement: Figure S4 — Hierarchical clustering of 85 CpG sites associated with the four HOX clusters. (TIF) [file pone.0016679.s004.tif]

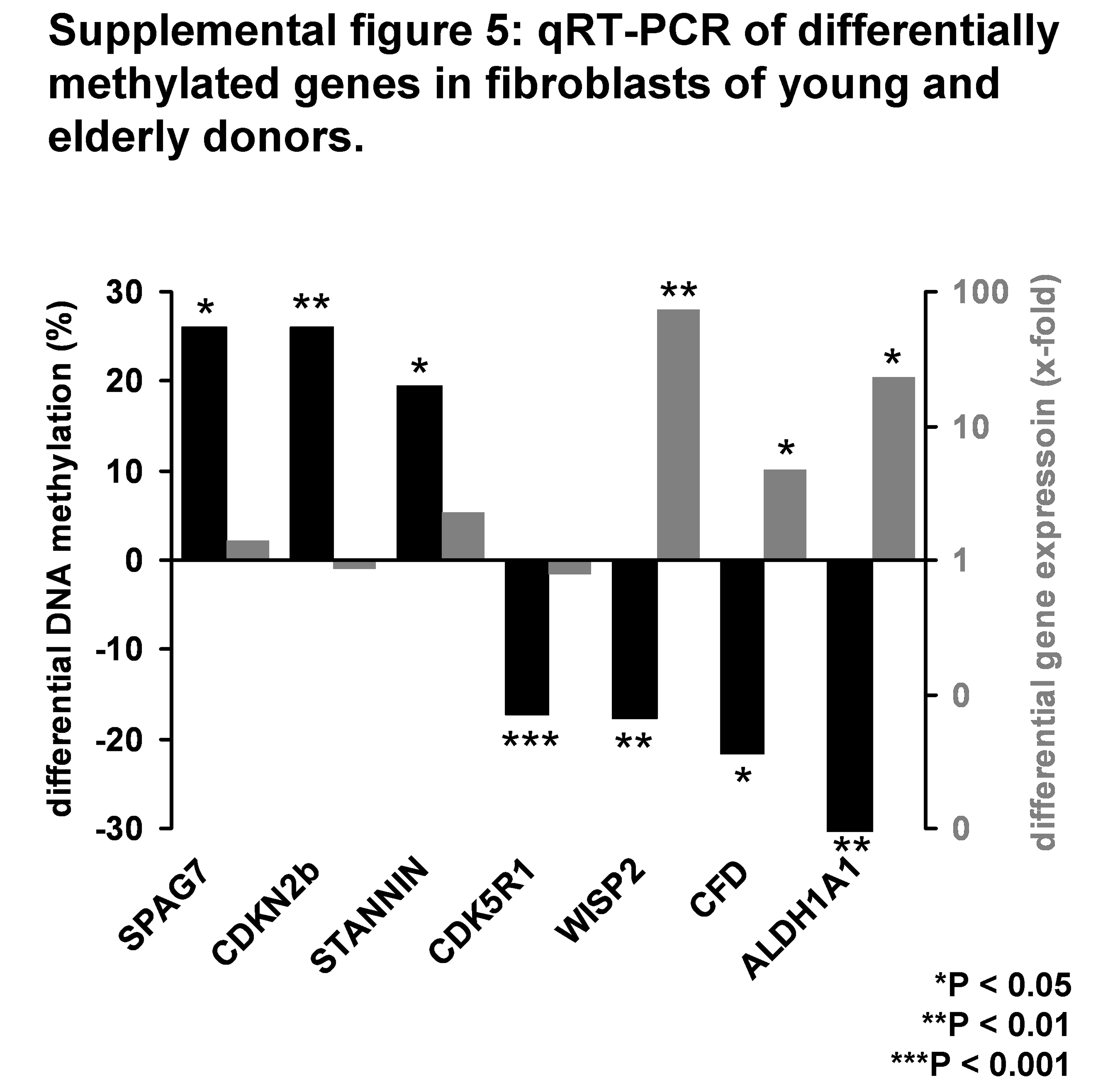

Supplement: Figure S5 — qRT-PCR of differentially methylated genes in fibroblasts of young and elderly donors. (TIF) [file pone.0016679.s005.tif]

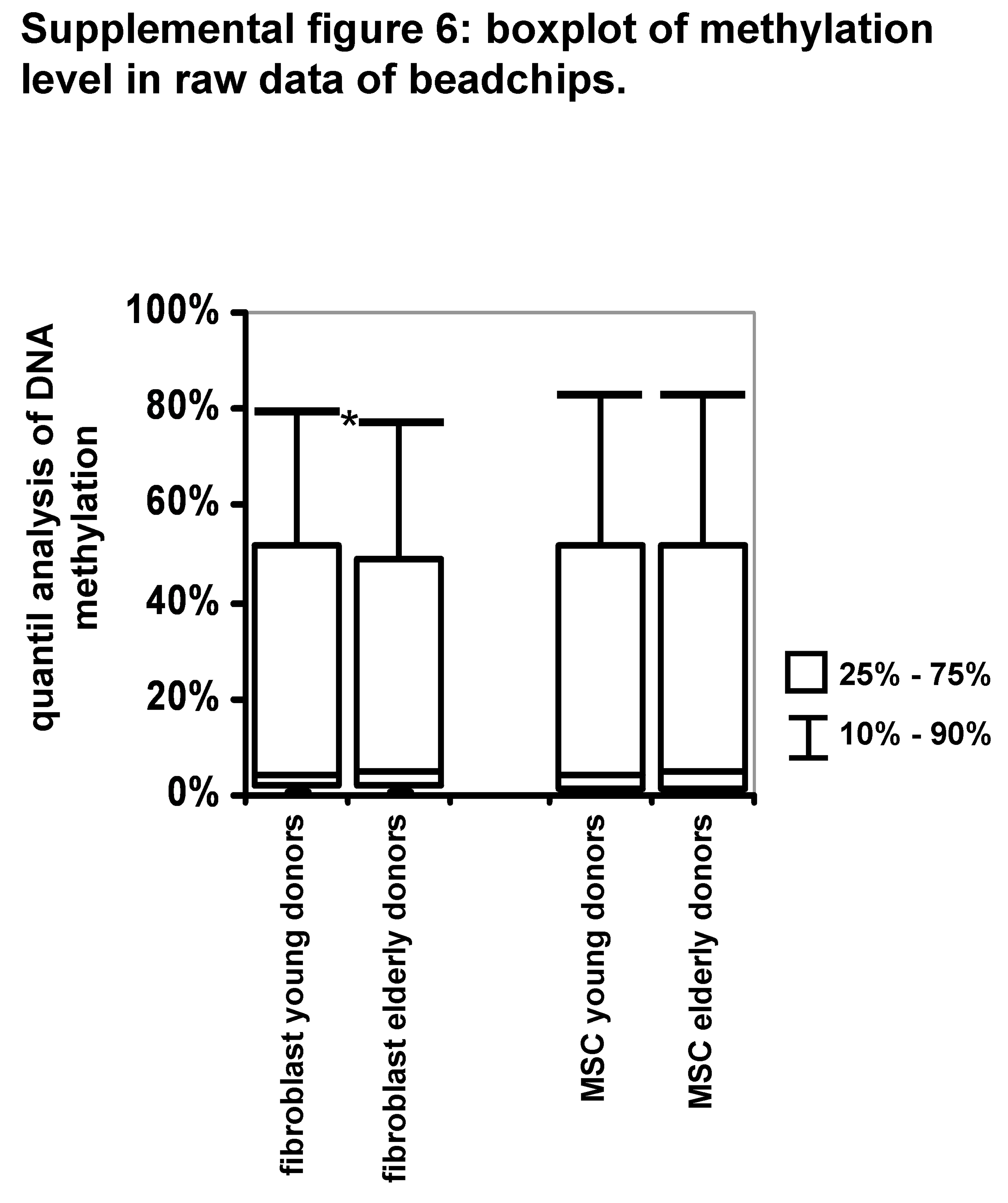

Supplement: Figure S6 — Boxplot of methylation level in raw data of beadchips. (TIF) [file pone.0016679.s006.tif]
