# Supplementary material for: Specific Age-Associated DNA Methylation Changes in Human Dermal Fibroblasts
Source: PLoS One. 2011 Feb 8;6(2):e16679. doi: 10.1371/journal.pone.0016679 (PMC3035656; doi:10.1371/journal.pone.0016679)
Supplement: Table S1 — Age-associated methylation changes in fibroblasts. (DOC) [file pone.0016679.s007.doc]

**Supplemental table 1: Age-associated methylation changes in fibroblasts.**

| **Target_ID** | **Symbol** | **Description** | **Methylation Change** | **RankProd adjusted**  **p-value** |
| --- | --- | --- | --- | --- |
| cg12815142 | SPAG7 | sperm associated antigen 7 | 26.1% | 0.036 |
| cg10210238 | CDKN2B | cyclin-dependent kinase inhibitor 2B isoform 2 | 26.0% | 0.001 |
| cg15308737 | ARSG | Arylsulfatase G | 19.6% | 0.036 |
| cg09816471 | SNN | Stannin | 19.5% | 0.024 |
| cg21184174 | NGFB | nerve growth factor; beta polypeptide precursor | 19.3% | 0.037 |
| cg03266453 | EN1 | engrailed homolog 1 | 18.8% | 0.019 |
| cg23178308 | C21orf124 | hypothetical protein LOC85006 | 17.2% | 0.014 |
| cg19497444 | SLC22A18 | tumor suppressing subtransferable candidate 5 | 16.9% | 0.000 |
| cg03294491 | SMAD2 | Sma- and Mad-related protein 2 | 16.5% | 0.009 |
| cg12621514 | DKK1 | dickkopf homolog 1 precursor | 16.2% | 0.030 |
| cg06458239 | ZNF549 | zinc finger protein 549 | 16.0% | 0.000 |
| cg14258236 | OR5V1 | olfactory receptor; family 5; subfamily V; member 1 | 15.3% | 0.042 |
| cg18515587 | SELENBP1 | selenium binding protein 1 | -15.7% | 0.004 |
| cg01346152 | DHRS3 | dehydrogenase/reductase (SDR family) member 3 | -15.8% | 0.006 |
| cg05342835 | SYNC1 | syncoilin; intermediate filament 1 | -16.0% | 0.006 |
| cg18939260 | MTSS1 | metastasis suppressor 1 | -16.5% | 0.001 |
| cg11471401 | KRT6A | keratin 6A | -16.7% | 0.001 |
| cg04106785 | CDK5R1 | cyclin-dependent kinase 5; regulatory subunit 1 | -17.2% | 0.000 |
| cg03562120 | WISP2 | WNT1 inducible signaling pathway protein 2 precursor | -17.7% | 0.002 |
| cg21057494 | CLEC3B | C-type lectin domain family 3; member B | -18.0% | 0.049 |
| cg26924825 | LCAT | lecithin-cholesterol acyltransferase precursor | -18.6% | 0.009 |
| cg12966875 | SLPI | secretory leukocyte peptidase inhibitor precursor | -18.6% | 0.000 |
| cg18680834 | ZNF536 | zinc finger protein 536 | -19.1% | 0.035 |
| cg19118077 | AKR1C3 | aldo-keto reductase family 1; member C3 | -19.3% | 0.008 |
| cg23081213 | PRKAG3 | AMP-activated protein kinase; non-catalytic gamma-3 subunit | -20.1% | 0.022 |
| cg11314271 | RGPD5 | RANBP2-like and GRIP domain containing 5 isoform 2 | -20.4% | 0.022 |
| cg26912636 | TMEPAI | transmembrane prostate androgen-induced protein isoform a | -20.8% | 0.012 |
| cg16601385 | CFD | complement factor D preproprotein | -21.7% | 0.050 |
| cg24169915 | FLJ25773 | hypothetical protein LOC283598 | -22.0% | 0.002 |
| cg21660392 | ABCA8 | ATP-binding cassette; sub-family A member 8 | -23.4% | 0.037 |
| cg16601861 | ALDH1A1 | aldehyde dehydrogenase 1A1 | -30.3% | 0.004 |
