# Supplementary material for: Specific Age-Associated DNA Methylation Changes in Human Dermal Fibroblasts
Source: PLoS One. 2011 Feb 8;6(2):e16679. doi: 10.1371/journal.pone.0016679 (PMC3035656; doi:10.1371/journal.pone.0016679)
Supplement: Table S2 — Age-associated changes in fibroblasts and MSC. (DOC) [file pone.0016679.s008.doc]

|  |  |  | **Fibroblasts** | | |  | **MSC** |  |
| --- | --- | --- | --- | --- | --- | --- | --- | --- |
| **Target_ID** | **Symbol** | **Description** | **mean young** | **mean old** | **Diff. meth.** | **mean young** | **mean old** | **Diff. meth.** |
| cg12815142 | SPAG7 | sperm associated antigen 7 | 37.8% | 64.0% | 26.1% | 43.8% | 17.6% | -26.2% |
| cg10210238 | CDKN2B | cyclin-dependent kinase inhibitor 2B isoform 2 | 28.5% | 54.4% | 26.0% | 60.3% | 43.6% | -16.7% |
| cg06144905 | PIPOX | L-pipecolic acid oxidase | 56.3% | 79.1% | 22.8% | 31.5% | 63.7% | 32.2% |
| cg13699808 | PRKCBP1 | protein kinase C binding protein 1 isoform a | 31.5% | 51.1% | 19.5% | 8.2% | 28.1% | 19.9% |
| cg21184174 | NGFB | nerve growth factor; beta polypeptide precursor | 32.8% | 52.1% | 19.3% | 58.8% | 36.3% | -22.5% |
| cg19103609 | PKN1 | protein kinase N1 isoform 2 | 41.9% | 59.5% | 17.6% | 41.1% | 68.8% | 27.7% |
| cg24816455 | SEMA3B | semaphorin 3B isoform 2 precursor | 38.8% | 54.9% | 16.1% | 8.0% | 27.1% | 19.1% |
| cg06458239 | ZNF549 | zinc finger protein 549 | 8.1% | 24.1% | 16.0% | 6.3% | 25.6% | 19.3% |
| cg15815843 | MFAP5 | microfibrillar associated protein 5 | 46.4% | 31.4% | -15.0% | 85.1% | 38.7% | -46.4% |
| cg13459560 | CSN3 | casein kappa | 56.1% | 41.1% | -15.0% | 27.9% | 44.2% | 16.3% |
| cg05485062 | SERPINA12 | serine (or cysteine) proteinase inhibitor; clade A (alpha-1 antiproteinase; antitrypsin); member 12 | 67.4% | 52.2% | -15.2% | 12.1% | 64.7% | 52.7% |
| cg07823492 | HOXB1 | homeo box B1 | 95.6% | 80.0% | -15.6% | 80.9% | 31.6% | -49.3% |
| cg18515587 | SELENBP1 | selenium binding protein 1 | 28.0% | 12.3% | -15.7% | 35.9% | 15.1% | -20.8% |
| cg01346152 | DHRS3 | dehydrogenase/reductase (SDR family) member 3 | 37.2% | 21.4% | -15.8% | 28.5% | 9.9% | -18.6% |
| cg05342835 | SYNC1 | syncoilin; intermediate filament 1 | 30.1% | 14.1% | -16.0% | 6.8% | 28.2% | 21.4% |
| cg06906435 | FLJ25773 | hypothetical protein LOC283598 | 65.0% | 48.9% | -16.2% | 5.1% | 47.6% | 42.5% |
| cg04245402 | C19orf21 | hypothetical protein LOC126353 | 40.3% | 24.0% | -16.3% | 51.1% | 26.3% | -24.8% |
| cg13144783 | CCR1 | chemokine (C-C motif) receptor 1 | 68.7% | 52.3% | -16.5% | 23.0% | 54.2% | 31.2% |
| cg03583857 | CD34 | CD34 antigen isoform b | 80.9% | 64.3% | -16.6% | 76.5% | 59.2% | -17.3% |
| cg23696886 | PDLIM2 | PDZ and LIM domain 2 isoform 3 | 52.1% | 34.8% | -17.3% | 3.7% | 47.6% | 43.9% |
| cg16177830 | TNFRSF17 | tumor necrosis factor receptor superfamily; member 17 | 51.5% | 34.0% | -17.6% | 57.5% | 76.3% | 18.8% |
| cg12347740 | MGC34647 | hypothetical protein LOC146433 | 54.1% | 36.5% | -17.6% | 87.2% | 59.5% | -27.7% |
| cg03562120 | WISP2 | WNT1 inducible signaling pathway protein 2 precursor | 29.3% | 11.6% | -17.7% | 34.1% | 10.6% | -23.5% |
| cg20398120 | PRKAG3 | AMP-activated protein kinase; non-catalytic gamma-3 subunit | 60.9% | 43.1% | -17.8% | 84.6% | 69.5% | -15.1% |
| cg15684563 | TSPAN8 | transmembrane 4 superfamily member 3 | 53.8% | 35.9% | -17.9% | 13.9% | 50.2% | 36.4% |
| cg23081213 | PRKAG3 | AMP-activated protein kinase; non-catalytic gamma-3 subunit | 44.2% | 24.1% | -20.1% | 49.8% | 29.7% | -20.1% |
| cg10664272 | CAPG | gelsolin-like capping protein | 56.1% | 36.0% | -20.1% | 96.4% | 77.1% | -19.3% |
| cg16601385 | CFD | complement factor D preproprotein | 52.2% | 30.4% | -21.7% | 15.1% | 37.4% | 22.2% |
| cg21660392 | ABCA8 | ATP-binding cassette; sub-family A member 8 | 51.8% | 28.4% | -23.4% | 42.8% | 17.8% | -25.0% |
| cg16992787 | CAPS | calcyphosine isoform b | 68.9% | 40.9% | -28.0% | 57.9% | 40.6% | -17.3% |
|  |  |  |  |  |  |  |  |  |
| This table summarizes CpG sites with more than 15% differential methylation in fibroblasts and MSC of young *versus* elderly donors. | | | | | | | | |

**Supplemental table 2: Age-associated changes in fibroblasts and MSC.**
