# Supplementary material for: Specific Age-Associated DNA Methylation Changes in Human Dermal Fibroblasts
Source: PLoS One. 2011 Feb 8;6(2):e16679. doi: 10.1371/journal.pone.0016679 (PMC3035656; doi:10.1371/journal.pone.0016679)
Supplement: Table S3 — Distribution of all the samples across the beadchips. (DOC) [file pone.0016679.s009.doc]

**Supplemental table 3: Distribution of all the samples across the beadchips**

| **Array** | **Sentrix Barcode** | **Sample** | **Tissue** | **Anatomical Site** | **Donor-Age** | **Age-Group** | **Passage** |
| --- | --- | --- | --- | --- | --- | --- | --- |
| **mesenchymal stromal cell data** | | | | | | | |
| 1 | 4439424008 | A | bone marrow | iliac crest | 25 yrs | young | 2 |
| 1 | 4439424008 | C | bone marrow | iliac crest | 25 yrs | young | 11 |
| 1 | 4439424008 | E | bone marrow | iliac crest | 24 yrs | young | 2 |
| 1 | 4439424008 | G | bone marrow | iliac crest | 24 yrs | young | 14 |
| 2 | 4439424075 | A | bone marrow | caput femoris | 79 yrs | old | 2 |
| 2 | 4439424075 | B | bone marrow | caput femoris | 85 yrs | old | 2 |
| 2 | 4439424075 | C | bone marrow | caput femoris | 79 yrs | old | 15 |
| 2 | 4439424075 | D | bone marrow | caput femoris | 85 yrs | old | 8 |
| 2 | 4439424075 | E | bone marrow | caput femoris | 85 yrs | old | 2 |
| 2 | 4439424075 | F | bone marrow | iliac crest | 50 yrs | young | 2 |
| 2 | 4439424075 | G | bone marrow | caput femoris | 85 yrs | old | 10 |
| 2 | 4439424075 | H | bone marrow | iliac crest | 50 yrs | young | 10 |
| 2 | 4439424075 | I | bone marrow | caput femoris | 53 yrs | old | 2 |
| 2 | 4439424075 | J | bone marrow | iliac crest | 21 yrs | young | 2 |
| 2 | 4439424075 | K | bone marrow | caput femoris | 53 yrs | old | 10 |
| 2 | 4439424075 | L | bone marrow | iliac crest | 21 yrs | young | 11 |
| **fibroblast data** | |  |  |  |  |  |  |
| 3 | 4811409040 | A | dermis | arm | 21 yrs | young | 3 |
| 3 | 4811409040 | B | dermis | arm | 63 yrs | old | 3 |
| 3 | 4811409040 | C | dermis | abdomen | 17 yrs | young | 3 |
| 3 | 4811409040 | D | dermis | abdomen | 73 yrs | old | 3 |
| 3 | 4811409040 | E | dermis | abdomen | 17 yrs | young | 3 |
| 3 | 4811409040 | F | dermis | abdomen | 63 yrs | old | 3 |
| 3 | 4811409040 | G | dermis | leg | 8 yrs | young | 3 |
| 3 | 4811409040 | H | dermis | leg | 61 yrs | old | 3 |
| 3 | 4811409040 | I | dermis | ear | 6 yrs | young | 3 |
| 3 | 4811409040 | J | dermis | eyelid | 64 yrs | old | 3 |
| 3 | 4811409040 | K | dermis | breast | 18 yrs | young | 3 |
| 3 | 4811409040 | L | dermis | breast | 60 yrs | old | 3 |
| 4 | 4811409047 | A | dermis | abdomen | 23 yrs | young | 3 |
| 4 | 4811409047 | C | dermis | ear | 6 yrs | young | 3 |
| 4 | 4811409047 | E | dermis | ear | 6 yrs | young | 21 |
