# Supplementary material for: Specific Age-Associated DNA Methylation Changes in Human Dermal Fibroblasts
Source: PLoS One. 2011 Feb 8;6(2):e16679. doi: 10.1371/journal.pone.0016679 (PMC3035656; doi:10.1371/journal.pone.0016679)
Supplement: Table S4 — Primer sequences for qRT-PCR. (DOC) [file pone.0016679.s010.doc]

**Supplemental table 4: Primer sequences for qRT-PCR.**

| **Gene** | **Product length (bp)** | **Forward Primer** | **Reverse Primer** |
| --- | --- | --- | --- |
| GAPDH | 110 | AATTGAGCCCGCAGCCTCC | CTTCCCCATGGTGTCTGAG |
| CDKN2B | 174 | AGTGGAGAAGGTGCGACAG | TCTACATCGGCGATCTAGG |
| SPAG7 | 467 | AAGACAGTGGGCAGATCAAG | CAGACGCTTCTTGGCTCTG |
| STANNIN | 521 | GCACACGGGACTGGTATTC | GGCCAAGTGGGTTTCAGAC |
| CDK5R1 | 118 | CAACGTGGTCTTCCTCTAC | GCCCATGTAGGAGTAGGAG |
| WISP2 | 192 | CAGCAGCTGTGAGGTGAAC | CACTCAGGGCAGCACTTGC |
| CFD | 89 | ATCAGCATGGGCCACGTAG | ACAAGCACCCACCTCCATG |
| ALDH1A1 | 137 | GGCAGTGAAGGCCGCAAGACA | ACTCCATTGTCGCCAGCAGCA |
